# Who is the ophthalmologist that developing countries need?

**DOI:** 10.5935/0004-2749.20210084

**Published:** 2021

**Authors:** Newton Kara-Junior

**Affiliations:** 1 Universidade de São Paulo, São Paulo, SP, Brazil

What makes a newly-graduated doctor choose a specialty? We need to have this discussion before defining who is the specialist we want to train to meet the social needs of developing countries.

We have observed that passion for a field of action is no longer the main reason for choosing a specialty. In general, favorable conditions in the job market and the possibility of combining a good quality of life with practice within the specialty are considerations that predominate in young graduates’ choices.

In Brazil, we believe there are many graduates who do not have the financial security to make a choice of specialty for meeting their long-term goals. The basic and immediate concerns of these graduates include obtaining a placement with a residency scholarship, flexibility of the curriculum (allowing them opportunity to work outside the program), and duration of the program; these factors may determine their choice of specialty.

With the objective of training specialists to meet the needs of society, we need well-trained ophthalmologists throughout the country of Brazil. To fulfill this objective, the target professionals (who are those most susceptible to the influences of public policy), in general, will be those who require satisfaction of their immediate needs as they specialize.

Inductive Policy of a state identifies the professionals required and guides their training. How might a specialist doctor in a large city choose to work in a place far from large urban centers? If they are a good practitioner, employers will want to keep them at the place where they completed medical residency, usually in a large city. Therefore, the government must create incentives for professionals to leave the capitals. The same reasoning applies for doctors from the countryside who go to the capitals to specialize; there must be incentives for their return. Because many places in the countryside have few of the structural conditions necessary for offering training, this may be a better solution than creating residency courses across the country.

In the case of ophthalmology, there are some particularities. Inductive Policy needs to offer stimulus for training of specialists who are generalists and who are willing to work in peripheral areas. As, in general, the place where specialization takes place is where professional relations are made, we believe that the most effective public policy is to encourage creation of good quality specialization courses in the countryside, with conditions that meet the needs of target professionals. Thus, public funding could be directed to regions with “assistance gaps” (regions that do not have eye care), and the number of vacancies offered could be proportionate to regional demand. A further potential Inductive Policy is hiring of professionals, after their specialization, by public health network in regions. This bridge between training and employment would offer conditions of security and predictability, to attract and hold professionals at desired locations.

It is important to differentiate between social and individual needs at this point. Many newly-graduated doctors will choose their specialization course as of convenience, and undertake their fellowship stage and work in large urban centers, as directed by their individual needs. There will be doctors who do not have the financial security to undertake specialization, who often work in primary care after their medical training. Inductive Policies could help young doctors to complete residency in ophthalmology, to meet social needs.

The state and professional leaders must articulate that satisfaction achieved through work occurs in a “win-win” equation, where both the specialist and society benefit.

In this context, another issue for discussion is whether the administration of the Medical Residency Program should be performed by the Ministry of Health, rather than the Ministry of Education. Medical residency is a unique postgraduate course; it is practical and with great appeal. Although an education perspective is important, as the resident is more a student than a worker, I believe that greater integration between both Ministries could bring specialization training closer to meeting social needs and also enable collaborative development of Integration Policies. In this scenario, the Ministry of Health could identify regions having “assistance gaps,” fund residency scholarships, and promote job opportunities after (and even during) specialization. The Ministry of Education could be responsible for establishing communication channels with academic and class leaders, who have the means to provide adequate teaching conditions for specialization courses, and to discuss joint teaching and assessment strategies.

In Hispanic Latin American countries, graduate doctors may work only in basic medical care or as specialists. However, in Brazil, doctors may work in any area of medicine; specialization is not mandatory for practicing in specialties. Should it be mandatory? In a medical clinic, the two-year internship is similar to residency, so the doctor is qualified to work as general practitioner. However, in ophthalmology, practice is not extensively taught during medical school training. Should specialization be mandatory in this case? We need to discuss which institutions can offer specialization and whether the title of Specialist should be mandatory.

We also need to think about whether three years of specialization in ophthalmology should be for all students. Three years of training can be too much for some and too little for others. Prof. Marcos Ávila thinks that if the student expects to work as an ophthalmologist, acting to meet society’s needs and in the public health system, probably two years of basic training would be sufficient, with an emphasis on refractometry and clinical ophthalmology. This would facilitate access for many newly-graduated doctors to this specialty and would optimize the resources of educational institutions. On the other hand, Prof. Paulo Augusto Mello considers it difficult to offer quality education in just two years, even if only basic training; perhaps this period would allow training a professional with little resolution capacity.

I believe that one of the problems in promoting public health in Brazil is that, although the doctor is not expensive per se, they are expensive for the system, because doctors without training for clinical reasoning often request excessive examinations and indicate treatments that do not work. If the ophthalmologist has poor resolution capacity, due to technical limitations resulting from incomplete training, their performance will be costly for the health system and for society^(1)^. Therefore, I believe that both experts cited above are correct in their assertions. However, I believe that the first step in training the ophthalmologists society needs should be to improve and standardize specialized training; secondly, to discuss the extent of basic specialization.
